# Mapping Functional Traits: Comparing Abundance and Presence-Absence Estimates at Large Spatial Scales

**DOI:** 10.1371/journal.pone.0044019

**Published:** 2012-08-31

**Authors:** Tim Newbold, Stuart H. M. Butchart, Çağan H. Şekercioğlu, Drew W. Purves, Jörn P. W. Scharlemann

**Affiliations:** 1 United Nations Environment Programme World Conservation Monitoring Centre, Cambridge, United Kingdom; 2 Computational Science Laboratory, Microsoft Research Cambridge, Cambridge, United Kingdom; 3 BirdLife International, Cambridge, United Kingdom; 4 Department of Biology, University of Utah, Salt Lake City, Utah, United States of America; Institute of Botany, Czech Academy of Sciences, Czech Republic

## Abstract

Efforts to quantify the composition of biological communities increasingly focus on functional traits. The composition of communities in terms of traits can be summarized in several ways. Ecologists are beginning to map the geographic distribution of trait-based metrics from various sources of data, but the maps have not been tested against independent data. Using data for birds of the Western Hemisphere, we test for the first time the most commonly used method for mapping community trait composition – overlaying range maps, which assumes that the local abundance of a given species is unrelated to the traits in question – and three new methods that as well as the range maps include varying degrees of information about interspecific and geographic variation in abundance. For each method, and for four traits (body mass, generation length, migratory behaviour, diet) we calculated community-weighted mean of trait values, functional richness and functional divergence. The maps based on species ranges and limited abundance data were compared with independent data on community species composition from the American Christmas Bird Count (CBC) scheme coupled with data on traits. The correspondence with observed community composition at the CBC sites was mostly positive (62/73 correlations) but varied widely depending on the metric of community composition and method used (R^2^: 5.6×10^−7^ to 0.82, with a median of 0.12). Importantly, the commonly-used range-overlap method resulted in the best fit (21/22 correlations positive; R^2^: 0.004 to 0.8, with a median of 0.33). Given the paucity of data on the local abundance of species, overlaying range maps appears to be the best available method for estimating patterns of community composition, but the poor fit for some metrics suggests that local abundance data are urgently needed to allow more accurate estimates of the composition of communities.

## Introduction

Efforts to describe the composition of communities have focused largely on the occurrence or abundance of species (e.g. [1], [2]). However, increasing attention is being paid to selected traits of organisms, such as body size or diet for animals, and maximum height and photosynthetic pathway for plants [3]. While a focus on the traits of organisms has a longer history in plant ecology, for example in dynamic global vegetation models (DGVMs; e.g. [4]), and in freshwater community ecology (e.g. [5]), it has only recently started to be adopted among animal ecologists (e.g. [6]–[9]).

A trait-based approach to community and ecosystem ecology is appealing for a number of reasons. First, the way that species respond to environmental changes is often related to certain traits [10]–[15], referred to as ‘functional response traits’ [16]. Second, the profile of other traits present in an ecosystem, referred to as ‘functional effect traits’ [16], is expected to determine the provision and rate of important ecological processes, such as plant productivity, pollination and seed dispersal [13], [17]–[19]. Finally, from a more practical perspective, the composition in terms of traits is probably easier to explain and predict than the particular species present in biological communities [3].

In response to this interest in traits, ecologists are beginning to generate maps of the trait composition of communities across large geographical areas [9], [20]–[24]. Useful metrics include the community-weighted mean, which is the mean trait value among all individuals in a community and can inform about the mechanisms driving community assembly [25], functional richness, which measures the range of trait values present in a community [26] and is linked to certain ecosystem processes [18], [27], and functional divergence, which measures the extent to which the individuals present in a community fill the available trait space [26] and is likely to be related to other ecosystem processes.

There are a number of challenges to mapping the trait composition of communities. One of the major challenges is accounting for the differing relative abundances of species in any one place. Previous attempts to describe biological communities in terms of the traits represented have often used species rather than individuals as the unit of analysis, thus ignoring differences in abundance among species (e.g. [6], [9], [13], [28], but see [8], [29]). In some cases it might be sufficient to know the distribution of trait values among the species present in any one place. However, when trying to understand the mechanisms underlying community assembly or the functioning of ecosystems, the number of *species* exhibiting a particular trait is likely to be less important than the number of *individuals*. The latter will be strongly affected by interspecific variation in local abundances, with the traits of the most common species being more strongly represented than those of rarer species. Species clearly differ widely in abundance and, if abundance varies systematically with the traits under consideration, will differ in their contribution to the profile of traits present and probably also in their contribution to ecosystem function.

Here, we investigate whether the accuracy and precision of maps of the trait composition of communities can be improved by incorporating estimates of local abundance interpolated from readily available abundance observations and estimates. We test for the first time the accuracy of maps of trait composition against observations. We focus on birds in the Western Hemisphere because good data on the abundance, distribution and traits of these species are available. Trait composition of communities was calculated from observed abundance of species from sites in the American Christmas Bird Count (CBC) scheme. For metrics of the trait composition of communities that include differences in the abundance of species, estimates made without abundance data will only be accurate if species with particular combinations of traits are not systematically more or less common than other species. We tested whether this was the case.

## Methods

### Data

#### Distribution maps

We inferred the non-breeding distributions of 4064 bird species that occur in the terrestrial Western Hemisphere (95% of the species known to occur in the region [30]) from extent of occurrence maps compiled by NatureServe [30]. We refined extent of occurrence maps by excluding elevations from a digital elevation model [31] outside the reported elevational limits in BirdLife International's World Bird Database (www.birdlife.org/datazone), to exclude areas which are unlikely to be inhabited and which otherwise would cause overestimation of species' distributions. After refinement, the distribution polygons were mapped as grids with 20×20 km resolution projected to a cylindrical equal-area WGS1984 (Behrmann) projection.

#### Trait data

We focused on four traits of bird species: body mass, generation length (the average age of breeding individuals), migratory behaviour and diet. These are likely to be functionally important: for example, bird diet is related to ecosystem functions such as pollination and seed dispersal [10], body size is important in determining food-web structure [32], and migratory behaviour may impose seasonality on any ecosystem functions performed by migrating birds. These traits represent only a subset of the many traits that might inform bird species' responses to global change or affect their role in ecosystem functioning; they were chosen as readily-available examples with which to evaluate the utility of different approaches for mapping functional traits.

Data on mass, generation length and migratory behaviour were taken from BirdLife International's World Bird Database. The data on body mass therein were compiled from various sources, primarily [33]. Data on generation length were based on published and unpublished estimates for age at first breeding, survival, and longevity, applied to the formulae recommended by the IUCN Standards and Petitions Subcommittee [34]. Where species-specific values of mass and generation length were not available (for 270 and 87 species, respectively), estimates were made based on mean values for congeners. As values of mass and generation length were highly right-skewed, they were log-transformed in all analyses.

Species were assigned to four migratory classes: non-migrants, nomads, altitudinal migrants, and latitudinal/longitudinal migrants. Nomadic species move in response to resources that are sporadic and unpredictable in distribution and timing, and may congregate, but not predictably in terms of location and timing. Altitudinal migrants regularly or seasonally make cyclical movements to higher or lower elevations with predictable timing and destinations. Latitudinal/longitudinal migrants are species for which a substantial proportion of the global or regional population makes regular or seasonal cyclical movements beyond the breeding range, with predictable timing and destinations. This includes species that may be migratory only in part of their range/population, short-distance migrants and migrants that occasionally respond to unusual conditions in a semi-nomadic way.

Diet data were compiled by one of us (CHS) from the literature, primarily from the *Handbook of the Birds of the World* ([35]; for a detailed description and a complete list of sources see [10]). Species were classified as having one of six diets: fruit, nectar, other plant material (including herbivorous omnivores), invertebrates, vertebrates, or mixed (including all other omnivores).

Complete trait data were available for 3960 species, including species for which mass and generation length values were interpolated from values for congeners. 104 species with incomplete trait data were excluded from our analyses to ensure consistency in the calculations across all methods of calculation and across all measures of community trait composition. Excluded species represented 33 different bird families and thus are unlikely to constitute a functionally unique set of species.

#### Abundance data

**Figure 1:**
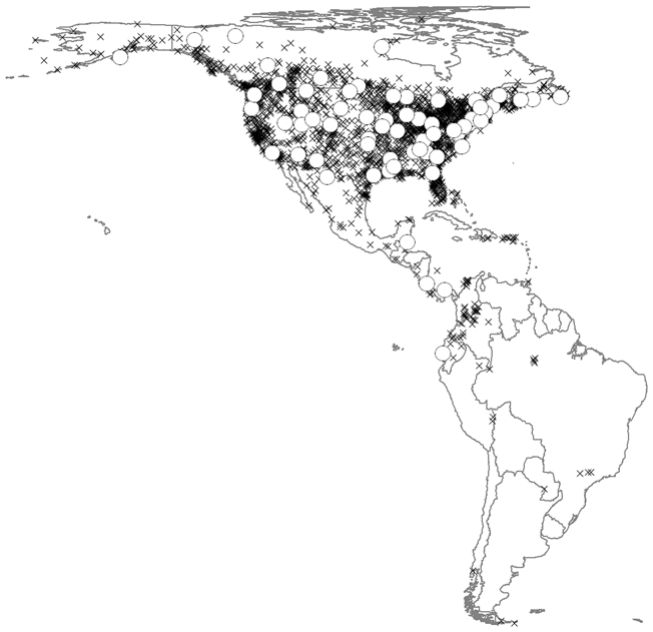
Sites with Christmas Bird Counts between 2000 and 2009. Black crosses are sites used in the generation of trait maps (n = 2398), whereas white circles are sites used for evaluating the maps (n = 68). In Behrmann cylindrical equal-area projection.

Observed abundance data for 2715 species were taken from 2466 sites (Figure 1) in the Christmas Bird Count (CBC) dataset, for ten years between 2000 and 2009 [36]. This dataset was chosen over other datasets, such as the North American Breeding Bird Survey, because of the larger spatial extent covered, including countries in Central and South America. CBC sites are almost entirely located in the northern hemisphere, with only 20 sites located south of the Equator. Counts were made each year on one day between 14th December and 5th January, with observers following specified routes within a circle of 24-km diameter [36]. The durations of the counts and number of people sampling differed among sites and years. Therefore, abundances were first corrected for sampling effort by dividing by the total duration of the count, then averaged across all years in which a given species was recorded at a site and rounded up to the nearest integer. Sites were split into training and evaluation subsets. To evaluate the accuracy of trait maps (see below), we reserved data from 68 CBC sites (henceforth ‘evaluation sites’) where sampling had occurred in at least five years. For evaluation sites, we used one site from each of the Canadian provinces, one from each of the United States, and one from each of the countries of Mexico, Belize, Costa Rica, Panama and Ecuador (see Figure 1). One of the evaluation sites was in the southern hemisphere. Pairwise distances between sites ranged from 23 to 9533 km (median: 2289 km). Thus, spatial autocorrelation should not present a major problem for the analyses. To ensure that this was the case, we repeated all analyses using spatial autoregressive models. The remaining 2398 CBC sites were used for developing the maps.

For one of the mapping methods, we also used estimates of the total global population size of bird species derived from BirdLife International's World Bird Database, available for 1324 of 3960 species. Estimates were taken from a wide variety of sources and were based on published estimates, surveys, censuses, inferences from distribution size, estimated population densities, and expert opinion. For species whose total population estimates consisted of minimum and maximum estimates, the mid-point of these values was taken. Because our study area was confined to the Western Hemisphere, we converted these estimates of global population size to estimates of total population size in the Western Hemisphere. To do this, we used a list of all countries in which each species was known to be resident or to overwinter. For each species, the global population estimate was reduced according to the ratio of the total area of these occupied countries in the Western Hemisphere to the total area of occupied countries in the whole world.

#### Environmental data

One of our mapping methods used three environmental variables: annual mean temperature and total annual precipitation, from the WorldClim dataset Version 1.4 [37], and absolute minimum temperature, calculated from WorldClim variables following Skov & Svenning [38]. Two additional environmental variables, growing degree days and water balance (*sensu* Skov & Svenning [38]), were considered initially but were excluded because they correlated strongly with annual mean temperature (R^2^ = 0.901 and 0.927 respectively).

### Mapping community trait composition

**Figure 2:**
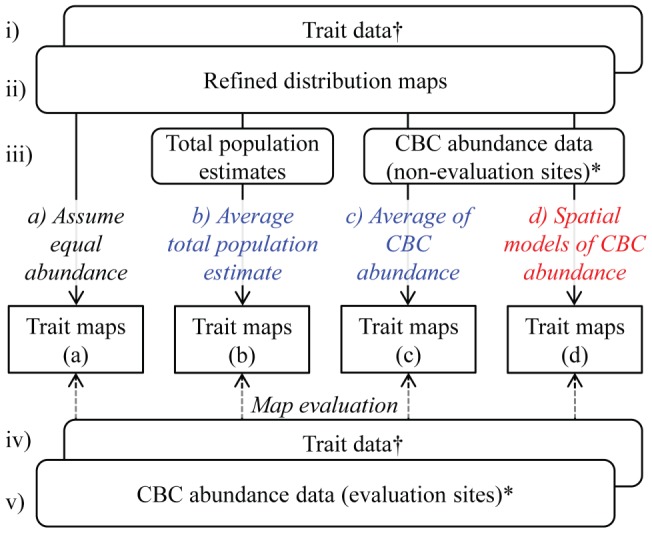
The basic scheme for generating and evaluating maps of the trait composition of bird communities. The maps were generated by combining i) trait data, ii) refined distribution maps and iii) various types of abundance data. We used four estimates of abundance (a–d) for generating the maps, based on three basic assumptions about the abundance of bird species: 1) that all species have an equal abundance (of one) in all grid cells (black text); 2) that species differ in abundance from one another, but with no spatial variation in abundance within species (blue text); and 3) that abundance varies both among species and spatially (red text). The maps were evaluated using iv) trait data and v) local abundance data from the Christmas Bird Count sites. Note (*) that the abundance data from the CBC sites were divided into a set for generating the maps (2398 sites) and a set for evaluating the resulting maps (68 sites). Note also (†) that the same trait data were used for generating and evaluating the maps.

Four methods were used to map species and trait composition based on the distribution maps and trait data (henceforth ‘distribution-based estimates’; Figure 2), making different assumptions about the relative abundance of species, thus requiring different amounts of data and accounting for different numbers of species: a) distribution maps for the 3960 species were overlaid, assuming that every species had the same abundance and that this was equal everywhere within each species' range; b) each species was assumed to be equally abundant throughout its range, but each species was allowed to differ in its abundance, estimated by dividing an estimate of each species' total population size in the Western Hemisphere (available for 1324 species) by its distribution area (number of grid cells in the distribution map); c) the same as method (b), but abundance for each species was estimated as the mean abundance of the species across the 2398 CBC sites not used for evaluation (data available for 2464 species); and d) abundances were allowed to vary both among species and within the distribution of each species, by modelling abundance at the 2398 non-evaluation CBC sites against three environmental variables (mean annual temperature, total annual precipitation and absolute minimum temperature) using generalized additive models fitting smoothing splines with 5 degrees of freedom. These models explained a high proportion (mean 60%, range 17–93%) of the variation in the abundance of the 503 species with at least 30 records in the CBC dataset. Each method included only those species for which the appropriate abundance data were available: a) n = 3960; b) n = 1324; c) n = 2449; d) n = 503 species. To check that the perceived relative accuracy of methods was not simply a result of the varying numbers of species, we recalculated the metrics for all methods including only the 351 species for which all of the methods could be applied (i.e. those species with complete distribution, abundance and trait data).

Using each of the four methods, for each trait individually and for all traits together, we generated maps of community-weighted mean trait values, functional richness and functional divergence. For categorical traits (migratory behaviour and diet), community weighted mean trait values were calculated as the proportion of individuals (or species) in each class. Functional richness was calculated as the range of trait values present for continuous traits and as the number of classes present for categorical traits. To calculate functional richness for all traits together, we first reduced the trait data of species using principal coordinates analysis (using the dudi.pco function in the ade4 package [39] in R Version 2.14.1 [40]), on a Gower-distance matrix of pairwise distances among species in trait space (using the gowdis function in the FD package [42] in R). We then calculated functional richness as the volume of a convex hull enclosing the principal coordinates of the species present in each grid cell (using the convhulln function in the geometry package [41] in R). Functional richness was only calculated using the method that ignored differences in species abundance, because functional richness measures by their nature cannot account for abundance. Functional divergence was calculated using the Rao index [26]:
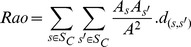
Where *A_s_* and *A_s'_* are the abundances of species *s* and *s'* in a community of total abundance *A* and containing *S_c_* species, and *d_(s,s')_* is the trait distance between species *s* and *s'*. Trait distances were calculated for each trait individually and for all traits together using Gower distances (using the gowdis function in the FD package [42] in R). Resulting values of the Rao diversity index were corrected to the numbers equivalent of the index following Jost [43], and as recommended by several authors as a more intuitive measure of diversity [44], [45], as follows:



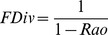



We chose to use the Rao index as a commonly used and easily calculated measure of functional divergence, rather than other available metrics such as those based on a ‘functional dendrogram’ [46], but whether our results hold for these other metrics is an important topic for further study.

We also generated a map of species richness, to test the ability of overlaid distribution maps to capture this basic measure of the composition of communities.

### Comparing observed and distribution-based estimates of metrics

Values of each of the trait composition metrics were calculated from local observed abundances at the CBC evaluation sites, and compared to the distribution-based estimates of the metrics using linear regression (see also Figure 2). We assessed whether local species composition at the evaluation sites was captured well by the range maps, by computing the Jaccard index of similarity between CBC-based and distribution-based estimates of species composition. This index varies from zero, if the local and distribution-based estimates share no species in common, to one, if the estimates are identical.

Ignoring differences in the abundance of species will only be a problem for mapping trait metrics if species' abundances in grid cells are biased with respect to the traits in question. For example if small-bodied species are more abundant on average than large-bodied species, then estimates of the distribution of trait values will be biased if it is assumed that species are equally abundant. On the other hand, if variations in abundance are random with respect to trait values, then estimates of the distribution of trait values should be unbiased. To test for a relationship between trait values and abundance, for each CBC site, we correlated log-transformed species abundance with body mass and generation length, and tested differences in (log-transformed) abundance with respect to migratory behaviour and diet using analysis of variance.

### Computational implementation and statistical analysis

All maps were generated using custom-built C# code developed in Microsoft® Visual Studio 10.0 (code available from TN on request). The precision of the distribution-based estimates was measured using R^2^ and the accuracy of the estimates by assessing departures from a fitted slope of zero. To test that the results were not influenced by spatial autocorrelation in the values of the metrics among CBC sites, the same analyses were repeated using simultaneous autoregressive (SAR) models. To do this, spatial weights were calculated based on the longitude and latitude coordinates of the evaluation sites using the tri2nb and nb2listw functions in the spdep package [47] in R. Spatial autoregressive models were then developed, using these weights, with the errorsarlm function in the spdep package. The results might be biased if species with particular combinations of traits are more easily detected than smaller species. To test this, we used a generalized linear model with a quasi-Poisson distribution of errors to relate the number of CBC sites at which a species was recorded to its (log-transformed) range size (measured as the number of 20-km grid cells in the refined range maps) and two traits hypothesized to have an effect on species' detectability: body mass and migratory behaviour.

## Results

The total local abundance of bird communities, estimated using the three mapping methods that incorporated abundance data, correlated positively with observed total local abundance at the Christmas Bird Count (CBC) sites, although the fit of these relationships varied depending on the method used (R^2^ values were: 0.34 where local abundance was estimated as a species' estimated total population size divided by range size; 0.52 where local abundance was estimated as the average abundance across CBC sites; and 0.57 where local abundance was modelled against environmental variables to capture spatial variation; see Table 1 for full results).

**Table 1:** Full results of the relationships between distribution-based and CBC-based estimates of community composition.

Trait	Metric	Method	Correlation		Departure from unity slope		
			R^2^	P	Slope	t	P
Mass	CWM	Range maps	**0.29**	**<0.001**	0.3	11.9	<0.001
		Total population	0.17	<0.001	**0.42**	**4.94**	**<0.001**
		Mean of records	0.19	<0.001	0.33	8.04	<0.001
		GAM models	0.17	<0.001	0.4	5.31	<0.001
	FRICH	Range maps	**0.55**	**<0.001**	**0.82**	**1.89**	**0.031**
	FDIV	Range maps	**0.0084**	**0.46**	−0.044	17.9	<0.001
		Total population	0.00048	0.86	**−0.017**	**10.5**	**<0.001**
		Mean of records	0.0013	0.77	−0.028	10.8	<0.001
		GAM models	0.0029	0.66	−0.064	7.26	<0.001
Generation length	CWM	Range maps	**0.31**	**<0.001**	0.42	7.42	<0.001
		Total population	0.25	<0.001	**1**	**0.109**	**0.46**
		Mean of records	0.17	<0.001	0.35	6.68	<0.001
		GAM models	0.17	<0.001	0.34	7.14	<0.001
	FRICH	Range maps	**0.48**	**<0.001**	**0.51**	**7.38**	**<0.001**
	FDIV	Range maps†	**0.097**	**0.0097**	0.22	9.19	<0.001
		Total population	0.041	0.099	0.29	4.18	<0.001
		Mean of records†	0.086	0.015	0.28	6.37	<0.001
		GAM models	0.14	0.0019	**0.41**	**4.76**	**<0.001**
Migratory behaviour	CWM (non-migratory)	Range maps	**0.54**	**<0.001**	**0.47**	**10.2**	**<0.001**
		Total population	0.032	0.14	0.26	4.32	<0.001
		Mean of records	0.035	0.12	0.2	6.12	<0.001
		GAM models†	0.084	0.018	−0.28	11.2	<0.001
	CWM (nomadic)	Range maps	0.38	<0.001	**1.2**	**1.22**	**0.11**
		Total population	**0.67**	**<0.001**	0.42	15.9	<0.001
		Mean of records	0.58	<0.001	0.43	12.4	<0.001
		GAM models	0.6	<0.001	0.52	8.96	<0.001
	CWM (altitudinal migrants)	Range maps	0.14	0.0016	**0.81**	**0.776**	**0.22**
		Total population	0.017	0.29	0.022	48	<0.001
		Mean of records	**0.22**	**<0.001**	0.56	3.4	<0.001
		GAM models	0.11	0.0066	0.28	7.33	<0.001
	CWM (full migrants)	Range maps	**0.54**	**<0.001**	**0.52**	**8**	**<0.001**
		Total population	0.036	0.12	0.27	4.34	<0.001
		Mean of records	0.039	0.11	0.21	6.19	<0.001
		GAM models†	0.075	0.024	−0.26	11.2	<0.001
	FRICH	Range maps	**0.36**	**<0.001**	**0.98**	**0.143**	**0.44**
	FDIV	Range maps	**0.53**	**<0.001**	**0.58**	**6.28**	**<0.001**
		Total population†	0.07	0.029	**−**0.25	11.2	<0.001
		Mean of records	0.004	0.61	**−**0.062	8.9	<0.001
		GAM models	0.025	0.2	**−**0.14	10.6	<0.001
Diet	CWM (fruit)	Range maps	**0.28**	**<0.001**	0.29	12.3	<0.001
		Total population	0.16	<0.001	0.23	11.7	<0.001
		Mean of records	0.089	0.014	**0.29**	**6.15**	**<0.001**
		GAM models	0.013	0.36	0.15	5.06	<0.001
	CWM (nectar)	Range maps	0.8	<0.001	**1.1**	**2.04**	**0.023**
		Total population	0.69	<0.001	0.69	5.52	<0.001
		Mean of records	**0.82**	**<0.001**	0.79	4.46	<0.001
		GAM models	0.77	<0.001	0.78	4.25	<0.001
	CWM (other plant material)	Range maps	**0.38**	**<0.001**	0.25	19.5	<0.001
		Total population†	0.068	0.032	0.27	5.96	<0.001
		Mean of records	0.12	0.0044	0.2	11.6	<0.001
		GAM models	0.23	<0.001	**0.42**	**6.06**	**<0.001**
	CWM (invertebrates)	Range maps†	0.3	<0.001	0.23	18	<0.001
		Total population	0.079	0.021	0.26	6.68	<0.001
		Mean of records	0.091	0.012	0.22	9.1	<0.001
		GAM models	**0.33**	**<0.001**	**0.59**	**3.86**	**<0.001**
	CWM (vertebrates)	Range maps	0.12	0.0034	0.17	14.6	<0.001
		Total population	0.011	0.39	0.1	7.84	<0.001
		Mean of records	0.45	<0.001	0.44	9.49	<0.001
		GAM models	**0.51**	**<0.001**	**0.93**	**0.629**	**0.27**
	CWM (mixed)	Range maps	0.0036	0.63	0.024	19.6	<0.001
		Total population	0.0065	0.51	**−**0.093	7.71	<0.001
		Mean of records	0.0064	0.52	0.048	12.8	<0.001
		GAM models†	**0.089**	**0.014**	**0.23**	**8.65**	**<0.001**
	FRICH	Range maps	**0.49**	**<0.001**	**0.58**	**5.69**	**<0.001**
	FDIV	Range maps	0.011	0.4	0.032	25.9	<0.001
		Total population	0.00055	0.85	0.015	12.4	<0.001
		Mean of records	0.00059	0.84	0.012	15.8	<0.001
		GAM models	**0.044**	**0.088**	**0.16**	**8.77**	**<0.001**
All traits	FRICH	Range maps	**0.45**	**<0.001**	**0.8**	**1.77**	**0.04**
	FDIV	Range maps	**0.18**	**<0.001**	**0.17**	**18.3**	**<0.001**
		Total population	0.0037	0.62	−0.054	9.71	<0.001
		Mean of records	5.7E-07	1	0.00052	11.8	<0.001
		GAM models	0.024	0.21	0.1	10.8	<0.001
Species richness		Range maps	**0.9**	**<0.001**	**0.99**	**0.332**	0.37
Total abundance		Total population	0.41	<0.001	0.22	24.1	<0.001
		Mean of records	0.34	<0.001	0.35	10.9	<0.001
		GAM models	**0.52**	**<0.001**	**0.4**	**12.5**	**<0.001**

For each of the four traits considered (mean mass, generation length, migratory behaviour and diet) and for all traits together, we calculated community-weighted mean trait values (CWM), functional richness (FRICH) and functional divergence (FDIV). The overall fit of the relationship between distribution-based estimates and observed community composition at the Christmas Bird Count sites was measured using a correlation test (R^2^ and P-values reported). Departures from a fitted relationship of y = x (i.e. a slope of 1) were assessed using a t-test (slope, t and P-values reported). For each metric the results of the strongest correlation and the smallest departure from one are shown in bold. †s indicate comparisons that were significant for these non-spatial models but that were non-significant for the spatial autoregressive models (for full spatial model results see Appendix S1).

**Figure 3:**
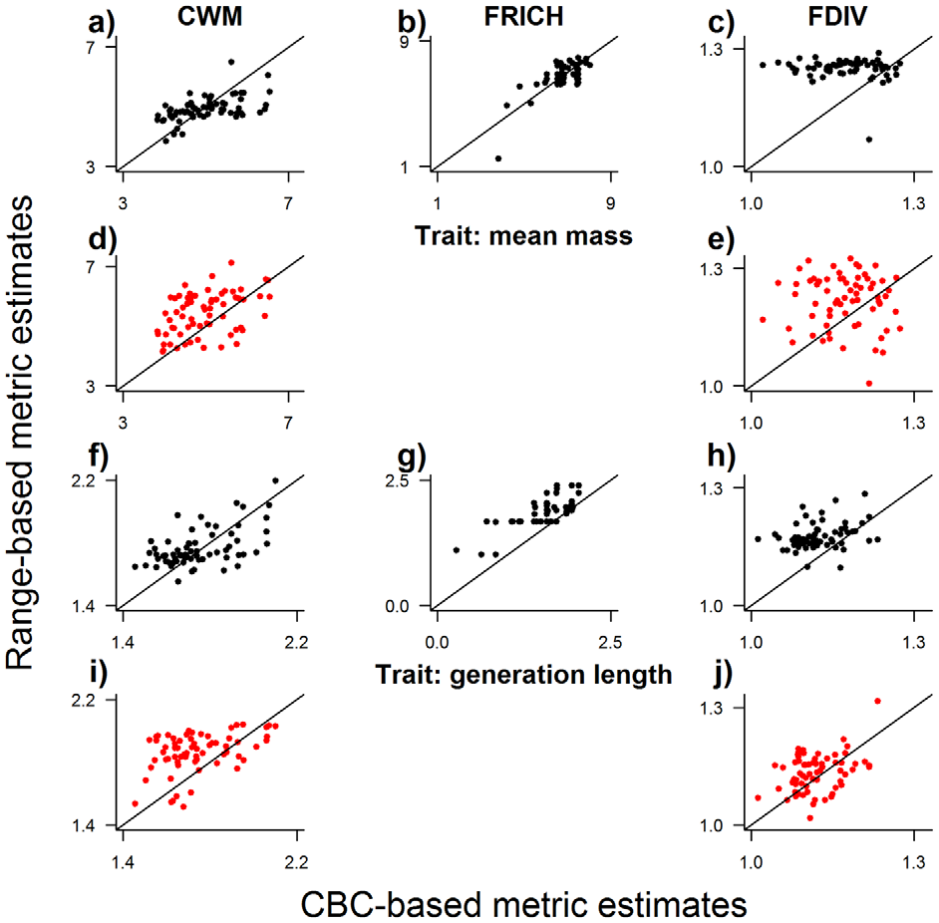
Correlation between distribution-based and CBC-based values of community composition metrics based on continuous traits. For each of the two continuous traits considered – body mass and generation length – maps were generated of community-weighted mean trait value (CWM; a, d, f, i), functional richness (FRICH; b, g) and functional divergence (FDIV; c, e, h, j). Observed values were calculated from recorded abundances at 68 Christmas Bird Count (CBC) evaluation sites. Distribution-based estimates of the metrics were generated using four methods, but only results from the best two methods are presented here: 1) overlaying range maps (black symbols); and 2) overlaying range maps with estimates of species abundance that vary among species and within species' ranges (red symbols). Abundance was estimated by modelling recorded abundances with respect to three environmental variables using generalized additive models. Lines represent y = x. Full results for all four methods are presented in Table 1.

In terms of precision (R^2^ values), the correspondence between distribution-based and CBC-based estimates of community trait composition varied widely (R^2^ from 5.7×10^−7^ to 0.82, median of 0.12), although most (62/73) relationships were positive (Figures 3–5; Table 1). Simply overlaying range maps and ignoring differences in local abundance produced the most precise estimates in 9 out of 17 cases (median R^2^ = 0.29), using total population size of each species to estimate local abundance gave the most precise estimate in 1 case (median R^2^ = 0.04), taking the average abundance at CBC sites for each species as an estimate of local abundance gave the most precise estimates in 2 cases (median R^2^ = 0.09), and using spatial models of local abundance gave the most precise estimates in 5 cases (median R^2^ = 0.11).

**Figure 4 pone-0044019-g004:**
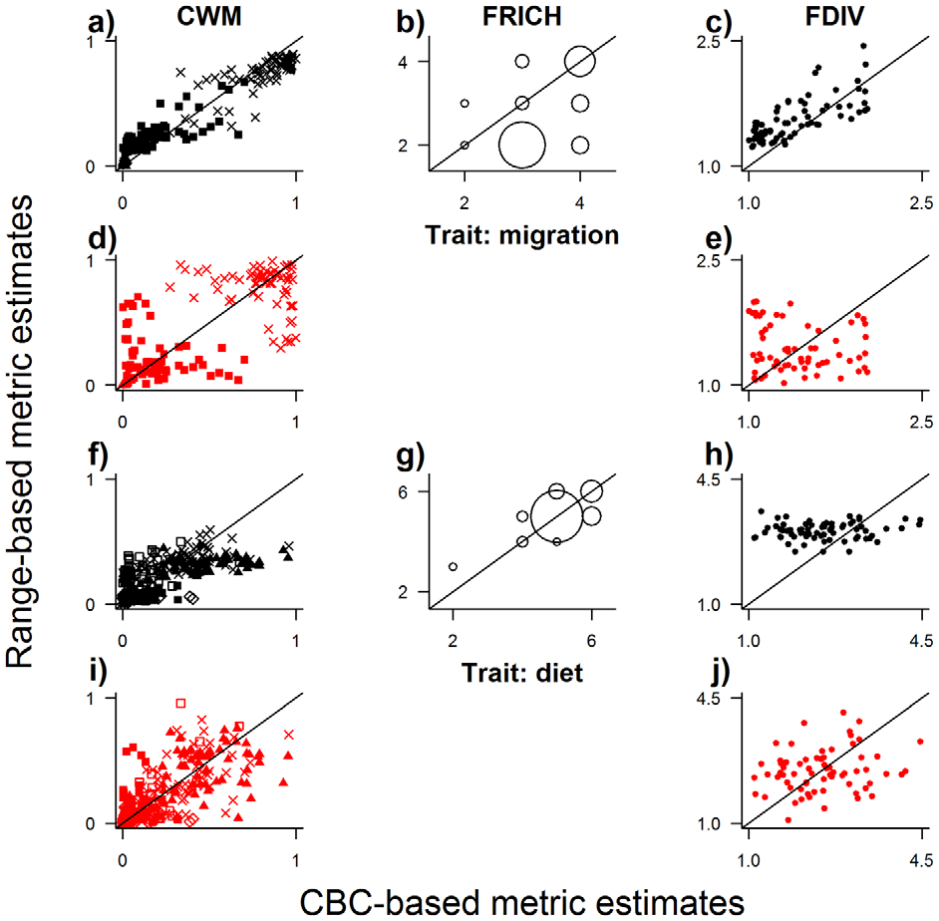
Correlation between distribution-based and CBC-based values of community composition metrics based on categorical traits. For each of the two categorical traits considered – migratory behaviour and diet – maps were generated of community-weighted mean trait value (CWM; a, d, f, i), functional richness (FRICH; b, g) and functional divergence (FDIV; c, e, h, j). For the categorical traits, community-weighted mean was calculated as the proportion of birds in each of the trait classes. Observed values were calculated from recorded abundances at 68 evaluation Christmas Bird Count (CBC) sites. Distribution-based estimates of the metrics were generated using four methods, with the best two shown here, as in Fig. 3. Lines represent y = x. Full results for all four methods are presented in Table 1.

**Figure 5:**
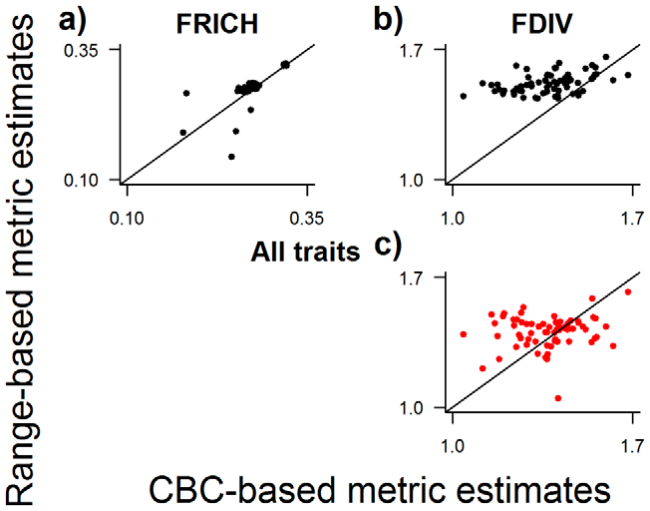
Correlation between distribution-based and CBC-based values of community composition metrics based on all traits together. Maps were generated of functional richness (FRICH; a) and functional divergence (FDIV; b, c). Functional divergence was measured using the Rao index. Observed values were calculated from recorded abundances at 68 evaluation Christmas Bird Count (CBC) sites. Distribution-based estimates of the metrics were generated using four methods, with the best two shown here, as in Fig. 3. Lines represent y = x. Full results for all four methods are presented in Table 1.

**Figure 6:**
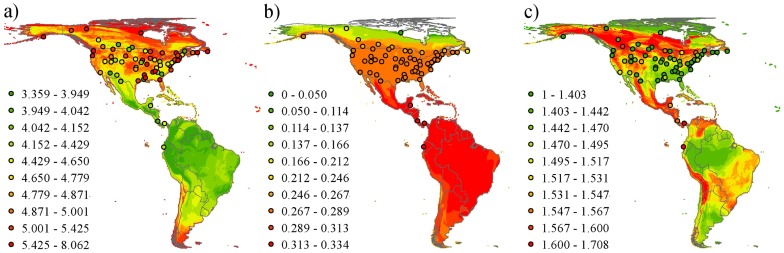
Examples of maps of the trait-based metrics with values at the CBC sites overlaid. a) community-weighted mean value of (log-transformed) body mass; b) functional richness based on all four functional traits (body mass, generation length, migratory behaviour and diet) measured as the volume of a convex hull enclosing all species positions in trait space; and c) functional divergence measured using the Rao index. Colour schemes for the rasters and for the points are the same. Displayed using the Behrmann cylindrical equal-area projection.

In terms of accuracy (approximation to a slope of one in the relationship between distribution-based and local estimates of the trait metrics), overlaying range maps was best in 7 out of 17 cases (mean slope estimate  = 0.40), using total population size of species was best in 3 cases (mean slope estimate  = 0.23), taking the average abundance of species at CBC sites was best in 1 case (mean slope estimate  = 0.25), and using spatial models of local abundance for each species was best in 6 cases (mean slope estimate  = 0.27; Figures 3, 4, 5; Table 1). For all methods, most relationships between distribution-based and CBC-based metrics had a slope that was significantly less than one. That is to say, the distribution-based estimates of community trait composition under-estimated observed community trait composition at higher observed values. Example maps are shown in Figure 6.

The map of species richness, generated by overlaying range maps, corresponded very closely with observed species richness at the CBC sites (R^2^ = 0.90; Table 1). However, the estimate of specific species composition generated by overlaying range maps did not always match the species composition estimated by the Christmas Bird Counts (Jaccard indices of compositional similarity ranged from 0.17 to 0.69, with a mean of 0.47).

Accounting for spatial autocorrelation by using SAR models did not alter the results substantially: in 8 out of 73 cases, the relationship was significant for the non-spatial models but non-significant for the spatial models (marked with a † in Table 1), but importantly the relative accuracy of the four methods was unchanged (for full spatial model results see Table S1, Appendix S1). The relative accuracy of the four mapping methods was not biased by the smaller number of species included in some methods: the results were very similar when all metrics were generated for only the 351 species with sufficient distribution, trait and abundance data for all four methods (Figures S1-S3 and Table S1, Appendix S2).

As expected, among bird species observed at CBC sites, there were more smaller-bodied species than larger-bodied species (Figure S4a, Appendix S3). However, the average local abundance of species was not strongly related to body mass (mean Pearson correlation coefficient across all sites was −0.025±0.003). Similarly, more species had shorter than longer generation length (Figure S4b, Appendix S3), but average recorded local abundance was not related to generation length (mean Pearson correlation coefficient across all sites was −0.016±0.003). Finally, local abundance did not generally differ with migratory behaviour (across all 2378 sites with sufficient data, analyses of variance revealed a significant difference – P<0.05 – for 55 sites, mean F = 1.04) or with diet (across 2383 sites with sufficient data, analyses of variance were significant for 7 sites, mean F = 3.10).

The apparent lack of correlations between the traits of species and their local abundances may reflect a failure to detect a relationship, arising from an effect of traits on the detectability of species and thus on estimated local abundance. This appeared to be the case to some extent: larger-bodied, migratory species with large range sizes were recorded at significantly more sites than small-bodied, non-migratory species with small range sizes. However, the effect of body mass on detectability was weak, with most of the variation in the number of sites at which a species was recorded being explained by range size and migratory behaviour (deviances explained: overall 46.7%; range size 19.7%; mass 3.9%; migratory behaviour 24.0%).

## Discussion

Previous attempts to map the distribution of traits and trait-based diversity metrics have involved overlaying distribution maps and have generally ignored differences in abundance among species [9], [20]–[24]. It will not always be necessary to account for abundance, but for some applications, such as understanding ecosystem processes and functioning, a consideration of abundance may be essential. It is not clear the extent to which estimates of trait-based metrics that do not account for abundance are a good proxy for observed values. This is the first time that the maps of the trait composition of communities have been compared with independent abundance data. We found that the correspondence between distribution-based estimates of community trait and species composition and the observed composition at the Christmas Bird Count evaluation sites varied widely and none of the four methods gave uniformly good results even for functional richness, which does not require abundance data for its calculation. This latter result was probably because overlaying range maps did not give an accurate estimate of species *composition* at all sites, even though estimates of species *richness* corresponded very well with species richness recorded in the Christmas Bird Counts. The low correspondence between estimates of species composition from range maps and observed species composition at the CBC sites was probably the result of two opposing effects: cases where the range maps predicted species to be present that were not detected were probably the result of the well-known over-prediction of extent-of-occurrence range maps [48], whereas species that were detected but not estimated to be present by the range maps were probably vagrants or scarce visitors. Nevertheless, for most metrics of trait composition, most estimates based on range maps and limited abundance data showed a positive relationship with estimates of the same metrics derived from the Christmas Bird Counts.

Importantly, for most metrics of community composition, the method of overlaying range maps produced nearly as good or better estimates of community composition, both in terms of accuracy and precision, than did the methods that included abundance, although even for this method the fit between distribution-based estimates and observed composition varied widely depending on the trait and metric considered. The fact that the methods that included abundance estimates failed to improve the estimated trait composition of communities was almost certainly because the methods used to estimate local abundance corresponded at best moderately with the abundance of bird communities observed in the Christmas Bird Counts (R^2^ values ranged from 0.34 to 0.52). In the following paragraphs, we will discuss the results for each of the community composition metrics separately.

Functional richness measures the range of different trait values present in a community and has been shown in some cases to correlate positively with measures of ecosystem functioning [18]. In this study, functional richness was generally the best estimated of the trait metrics considered, both for individual traits and for all traits together (Table 1). This is probably because its calculation relies only on an estimate of species composition and does not involve any assumption about abundance. The lack of a perfect match between the distribution-based and CBC-based estimates of functional richness was because species composition at the CBC sites was imperfectly estimated by overlaying range maps. This mismatch in species composition estimates explains to some degree the mismatch between the distribution-based and CBC-based estimates of all of the other metrics.

The trait composition of communities can also be measured as the mean trait value of species in the community, weighted by those species’ relative abundances (community-weighted mean trait values; e.g. [19]). These community-weighted mean trait values have been shown to be associated with rates of important ecosystem processes [19] and with environmental gradients [49]. In this study, estimates of the community-weighted mean values of the two continuous traits considered, made by overlaying range maps and ignoring abundance, showed a positive albeit relatively weak correlation with observed values at the CBC sites, which accounted for differences in abundance (R^2^ values of 0.29 and 0.31; Figure 3a and 3f). Estimates of the proportion of individuals in a community exhibiting a particular migratory behaviour or belonging to a particular diet guild also correlated positively in every case with observed values from the CBC sites, although R^2^ values varied widely (0.003 to 0.8; Figure 3). The additional mismatch between distribution-based and CBC-based estimates of community-weighted mean trait values, beyond that for functional richness, suggests that more accurate estimates of abundance could improve the distribution-based estimates. However, none of the methods tested here for estimating local abundances gave results that were better than simply overlaying range maps.

The functional trait composition of communities can also be measured as the extent to which individuals in a community fill trait space (functional divergence). As with community-weighted mean trait values and functional richness, it has been suggested that functional divergence is related to the functioning of ecosystems and the delivery of ecosystem services [9], [16], [50]. In this study, even with the best method, estimates of functional divergence based on all four traits showed a relative weak association with observed values from the CBC sites. This appeared to be owing to the fact that the range of values estimated was much lower than the range of values observed at the CBC sites (Figure 4), suggesting that much greater variation in functional divergence is apparent when accounting for differences in species abundance than when only species identity is considered. Functional divergence with respect to individual traits was even more poorly estimated, indeed showing virtually no correspondence at all with observed communities. As for functional divergence based on all traits, estimated values typically spanned a much smaller range than observed values. To some extent this is expected because local observations of abundance are subject to additional sources of variation including interannual variation in species abundance, environmental heterogeneity not captured at the resolution of the maps, and measurement error (e.g. failure to detect species). Nevertheless, the results suggest that it will be very difficult to capture information about the variability in the functional traits represented in biological communities accurately without detailed information on the abundance of each species. Nevertheless, as with community-weighted mean trait values, none of the methods tested here for estimating local abundances gave better results than simply overlaying range maps and ignoring differences in abundance.

Given that in any one location species are known to differ greatly in abundance, it is perhaps surprising that overlaying distribution maps gave the maps of community trait composition that showed the best fit to observed trait composition. However, for the purposes of mapping trait distributions, ignoring differences in abundance will only be a problem if the average local abundance of species is related to the traits under consideration. For example, it has been shown that body mass often correlates with abundance [51]. However, for sites in the Christmas Bird Count, while there were a greater number of small-bodied than large-bodied bird *species* recorded, the average local abundance of small species was not greater than that of large-bodied species. That is to say, the observation that sites tend to contain a greater number of small than large *individuals* is accounted for by there being a greater number of small than large species. We also found no evidence of a relationship between average local abundance and the other traits considered. A relationship between traits and average local abundance might have been masked if the traits had a strong effect on species detectability. This was the case for some of the traits considered: large-bodied, migratory species with large range sizes were observed at significantly more CBC sites than small-bodied non-migratory species with small range sizes, although the effect of body mass was weak. Probability of detection in birds is also related to conspicuousness of vocalisations [52] and species' specialisation [53] but we did not consider these traits here.

Finally, a metric that is not trait based, but will continue to be of interest to ecologists, is local species richness. We found that the maps of species richness produced by the distribution overlap method correlated strongly with observed species richness at the evaluation sites (Table 1). It has been shown by others (e.g. [48]) that overlaying maps of the extent of occurrence of species tends to overestimate local species richness owing to errors of commission. However, this was not the case here (Figure 5), probably because we refined distributions excluding areas outside the recorded elevational limits of each species. It is interesting to note that while species richness was very well estimated, the species composition estimated by overlaying range maps did not correspond particularly well with the species composition observed at all CBC sites.

It is important to note that this study considered only one taxonomic group. Furthermore, the abundance data used covered the northern hemisphere more extensively than the southern hemisphere, and the range maps are likely to be more accurate for North America than for South America. Therefore, caution is needed in interpreting the estimates of community trait composition for the southern part of the study area considered here. Further work is needed to test whether our results apply to regions outside North America and for other taxonomic groups. Overall though, it would seem that, given more numerous and geographically wide-ranging data on the abundance of a greater proportion of species, it might be possible to improve the estimation and mapping of abundance and traits, but such data are lacking at present even for birds, which are among the best studied of the taxonomic groups. However, in the absence of detailed abundance data, overlaying distribution maps appears to be the best method available at present and produced apparently accurate maps for at least some of the metrics of community trait composition considered here.

## Supporting Information

Appendix S1 **Results of simultaneous autoregressive (SAR) models of the relationships between distribution-based and Christmas Bird Count-based estimates of community composition.**
(DOC)

Appendix S2 **For species with complete distribution, trait and abundance data, correlations between distribution-based and Christmas Bird Count-based values of the community abundance and trait composition metrics.**
(DOC)

Appendix S3 **Frequency distributions of species with respect to body mass and generation length.**
(DOC)
