# Supplementary material for: Mapping Functional Traits: Comparing Abundance and Presence-Absence Estimates at Large Spatial Scales
Source: PLoS One. 2012 Aug 31;7(8):e44019. doi: 10.1371/journal.pone.0044019 (PMC3432103; doi:10.1371/journal.pone.0044019)
Supplement: Appendix S1 — Results of simultaneous autoregressive (SAR) models of the relationships between distribution-based and Christmas Bird Count-based estimates of community composition. (DOC) [file pone.0044019.s001.doc]

**Appendix S1. Results of simultaneous autoregressive (SAR) models of the relationships between range-based and Christmas Bird Count-based estimates of community composition**. To check that comparison of map accuracy among different methods was not influenced by spatial autocorrelation in the community composition metrics at the CBC sites.

**Table S1. Results of simultaneous autoregressive (SAR) models of the relationships between range-based and Christmas Bird Count-based estimates of community composition**.

| Trait | Metric | Method | Slope | z | P | Pseudo-R^2^ |
| --- | --- | --- | --- | --- | --- | --- |
| Mass | CWM | Range maps | 0.25 | 4.7 | < 0.001 | 0.39 |
|  |  | Total population | 0.32 | 3.2 | 0.0013 | 0.39 |
|  |  | Mean of records | 0.29 | 3.5 | < 0.001 | 0.23 |
|  |  | GAM models | 0.33 | 3.2 | 0.0015 | 0.25 |
|  | FRICH | Range maps | 0.76 | 8.1 | < 0.001 | 0.6 |
|  | FDIV | Range maps | -0.046 | -0.8 | 0.42 | 0.024 |
|  |  | Total population | -0.026 | -0.29 | 0.77 | 0.067 |
|  |  | Mean of records | -0.041 | -0.45 | 0.65 | 0.066 |
|  |  | GAM models | -0.097 | -0.81 | 0.42 | 0.22 |
| Generation length | CWM | Range maps | 0.39 | 5.2 | < 0.001 | 0.39 |
|  |  | Total population | 0.92 | 5 | < 0.001 | 0.47 |
|  |  | Mean of records | 0.35 | 3.6 | < 0.001 | 0.17 |
|  |  | GAM models | 0.29 | 3.2 | 0.0016 | 0.21 |
|  | FRICH | Range maps | 0.48 | 6.7 | < 0.001 | 0.51 |
|  | FDIV | Range maps | 0.12 | 1.6 | 0.1 | 0.28 |
|  |  | Total population | 0.18 | 1.4 | 0.15 | 0.44 |
|  |  | Mean of records | 0.16 | 1.4 | 0.15 | 0.12 |
|  |  | GAM models | 0.23 | 2 | 0.044 | 0.26 |
| Migratory behaviour | CWM  (non-migratory) | Range maps | 0.42 | 7.7 | < 0.001 | 0.57 |
|  |  | Total population | 0.14 | 0.84 | 0.4 | 0.2 |
|  |  | Mean of records | 0.21 | 1.7 | 0.095 | 0.21 |
|  |  | GAM models | -0.03 | -0.34 | 0.74 | 0.49 |
|  | CWM  (nomadic) | Range maps | 1.1 | 6.1 | < 0.001 | 0.49 |
|  |  | Total population | 0.37 | 10 | < 0.001 | 0.71 |
|  |  | Mean of records | 0.35 | 8.6 | < 0.001 | 0.66 |
|  |  | GAM models | 0.42 | 8.1 | < 0.001 | 0.63 |
|  | CWM  (altitudinal migrants) | Range maps | 0.86 | 4.9 | < 0.001 | 0.5 |
|  |  | Total population | 0.015 | 0.79 | 0.43 | 0.13 |
|  |  | Mean of records | 0.57 | 4.7 | < 0.001 | 0.29 |
|  |  | GAM models | 0.27 | 3.2 | 0.0014 | 0.29 |
|  | CWM  (full migrants) | Range maps | 0.46 | 7.4 | < 0.001 | 0.57 |
|  |  | Total population | 0.16 | 0.95 | 0.34 | 0.2 |
|  |  | Mean of records | 0.22 | 1.8 | 0.075 | 0.22 |
|  |  | GAM models | -0.0093 | -0.11 | 0.92 | 0.49 |
|  | FRICH | Range maps | 0.5 | 3.7 | < 0.001 | 0.58 |
|  | FDIV | Range maps | 0.51 | 7.4 | < 0.001 | 0.54 |
|  |  | Total population | -0.081 | -0.72 | 0.47 | 0.16 |
|  |  | Mean of records | 0.084 | 0.82 | 0.41 | 0.3 |
|  |  | GAM models | 0.13 | 1.7 | 0.081 | 0.48 |
| Diet | CWM  (fruit) | Range maps | 0.25 | 5.8 | < 0.001 | 0.5 |
|  |  | Total population | 0.23 | 4.2 | < 0.001 | 0.33 |
|  |  | Mean of records | 0.31 | 2.9 | 0.0042 | 0.15 |
|  |  | GAM models | 0.17 | 1.2 | 0.23 | 0.21 |
|  | CWM  (nectar) | Range maps | 1.1 | 14 | < 0.001 | 0.81 |
|  |  | Total population | 0.66 | 11 | < 0.001 | 0.71 |
|  |  | Mean of records | 0.79 | 17 | < 0.001 | 0.82 |
|  |  | GAM models | 0.79 | 17 | < 0.001 | 0.78 |
|  | CWM  (other plant material) | Range maps | 0.13 | 3.9 | < 0.001 | 0.56 |
|  |  | Total population | 0.21 | 1.7 | 0.091 | 0.15 |
|  |  | Mean of records | 0.22 | 3.2 | 0.0015 | 0.17 |
|  |  | GAM models | 0.26 | 3.1 | 0.0023 | 0.42 |
|  | CWM  (invertebrates) | Range maps | 0.035 | 1.2 | 0.24 | 0.67 |
|  |  | Total population | 0.24 | 2.1 | 0.036 | 0.089 |
|  |  | Mean of records | 0.24 | 3.1 | 0.0018 | 0.11 |
|  |  | GAM models | 0.3 | 3.4 | < 0.001 | 0.59 |
|  | CWM  (vertebrates) | Range maps | 0.11 | 2.2 | 0.03 | 0.43 |
|  |  | Total population | 0.061 | 0.56 | 0.58 | 0.29 |
|  |  | Mean of records | 0.28 | 4.8 | < 0.001 | 0.57 |
|  |  | GAM models | 0.89 | 7.8 | < 0.001 | 0.51 |
|  | CWM  (mixed) | Range maps | 0.012 | 0.27 | 0.79 | 0.21 |
|  |  | Total population | -0.073 | -0.53 | 0.6 | 0.05 |
|  |  | Mean of records | 0.03 | 0.42 | 0.68 | 0.06 |
|  |  | GAM models | 0.083 | 1.1 | 0.28 | 0.23 |
|  | FRICH | Range maps | 0.59 | 8.3 | < 0.001 | 0.58 |
|  | FDIV | Range maps | 0.0084 | 0.3 | 0.76 | 0.34 |
|  |  | Total population | 0.017 | 0.22 | 0.83 | 0.018 |
|  |  | Mean of records | 0.039 | 0.69 | 0.49 | 0.06 |
|  |  | GAM models | 0.1 | 1.2 | 0.22 | 0.19 |
| All traits | FRICH | Range maps | 0.38 | 4.9 | < 0.001 | 0.67 |
|  | FDIV | Range maps | 0.14 | 3.5 | < 0.001 | 0.26 |
|  |  | Total population | -0.033 | -0.31 | 0.75 | 0.036 |
|  |  | Mean of records | -0.016 | -0.21 | 0.83 | 0.16 |
|  |  | GAM models | 0.13 | 1.8 | 0.076 | 0.16 |
| Species richness | | Range maps | 0.82 | 17 | < 0.001 | 0.91 |
| Total abundance | | Total population | 0.13 | 2.5 | 0.012 | 0.57 |
|  | | Mean of records | 0.22 | 4.9 | < 0.001 | 0.64 |
|  | | GAM models | 0.47 | 6.8 | < 0.001 | 0.65 |

Four methods were used to generate the maps: 1) overlaying range maps, thus ignoring differences in species abundance (‘Range maps’); 2) assuming equal abundance throughout species’ ranges but allowing species to differ in their average grid cell abundance, estimated by dividing estimates of total population size by distribution area (‘Total population’); 3) as in the previous method but estimating species’ grid cell abundances as the average recorded abundance at the 2398 non-evaluation CBC sites (‘Mean of records’); and 4) allowing species abundance to vary among species and within ranges, with abundance estimated across the study area by modelling recorded abundances with respect to three environmental variables using generalized additive models (‘GAM models’). For each of the four traits considered (mean mass, generation length, migratory behaviour and diet) and for all traits together, we calculated community-weighted mean trait values (CWM), functional richness (FRICH) and functional divergence (FDIV). The fit of the relationship was assessed using z values and associated P values, and Nagelkerke's pseudo-R^2^. All of these analyses were carried out using the spdep package [1] in R Version 2.14.2 [2].

# REFERENCES

1. Bivand R (2012) spdep: Spatial dependence: weighting schemes, statistics and models. R Package Version 0.5-46. p. Available:http://cran.r-project.org/web/packages/spdep.

2. R Development Core Team (2010) R: A Language and Environment for Statistical Computing. Vienna, Austria: R Foundation for Statistical Computing. p. Available:http://www.r-project.org.
