# Supplementary material for: Mapping Functional Traits: Comparing Abundance and Presence-Absence Estimates at Large Spatial Scales
Source: PLoS One. 2012 Aug 31;7(8):e44019. doi: 10.1371/journal.pone.0044019 (PMC3432103; doi:10.1371/journal.pone.0044019)
Supplement: Appendix S2 — For species with complete distribution, trait and abundance data, correlations between distribution-based and Christmas Bird Count-based values of the community abundance and trait composition metrics. (DOC) [file pone.0044019.s002.doc]

**Appendix S2. For species with complete distribution, trait and abundance data, correlations between range-based and Christmas Bird Count-based values of the community abundance and trait composition metrics**. Maps were generated including only the 351 species with complete distribution, abundance and trait data.

**Figure S1. Correlation between range-based and Christmas Bird Count-based values of community composition metrics based on continuous traits.** For the 351 species with complete distribution, trait and abundance data. For each of the two continuous traits considered – body mass and generation length – maps were generated of community-weighted mean trait value (CWM; a, d, f, i), functional richness (FRICH; b, g) and functional divergence (FDIV; c, e, h, j). Observed values were calculated from recorded abundances at 68 Christmas Bird Count (CBC) evaluation sites. Modelled values of the metrics were generated using four methods, but only results from the best two methods are presented here: 1) overlaying range maps (black symbols); and 2) allowing species abundance to vary among species and within ranges, with abundance estimated across the study area by modelling recorded abundances with respect to three environmental variables using generalized additive models (red symbols). Lines represent y = x. Full results for all four methods are presented in Table S2 (below).


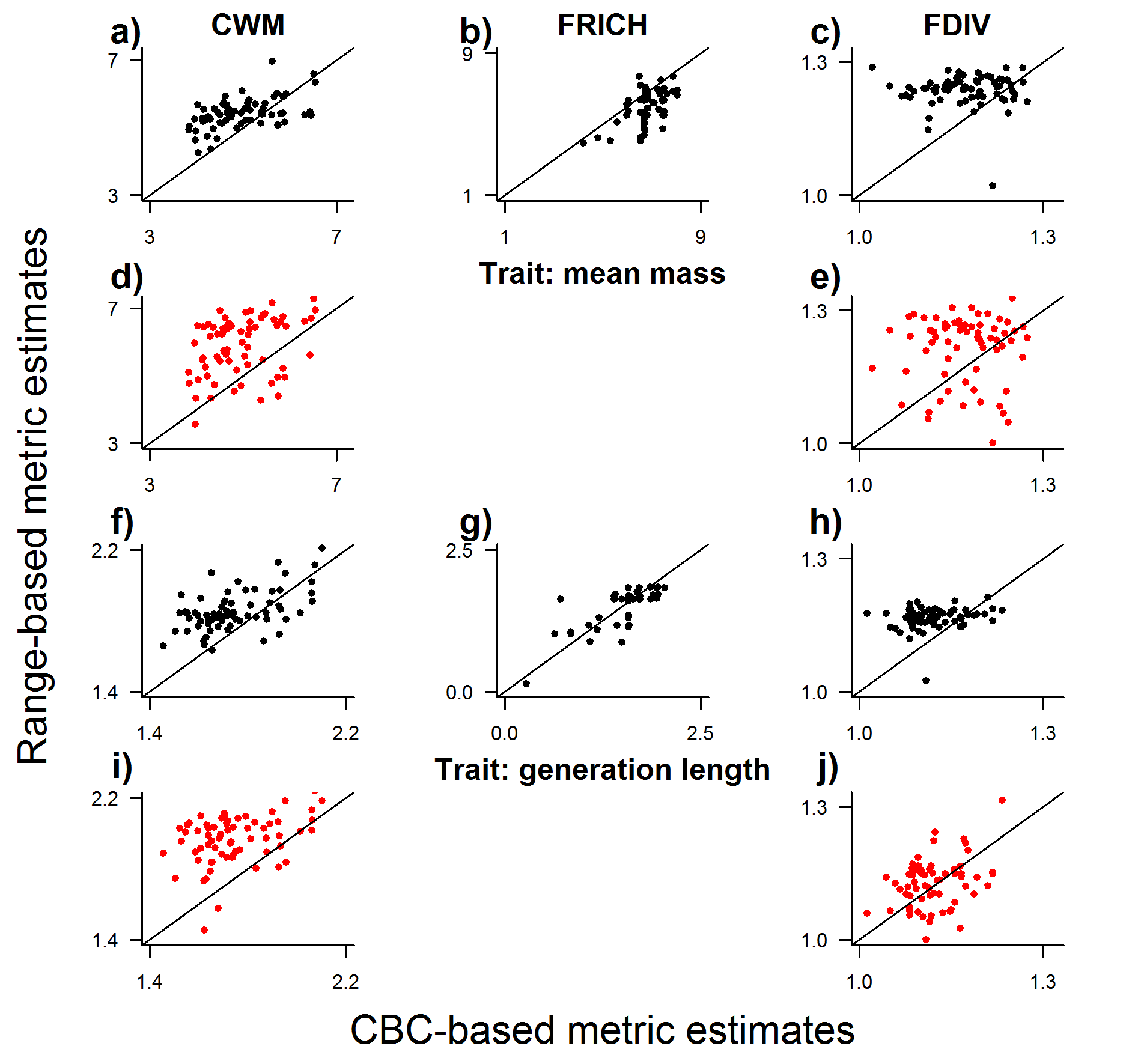


**Figure S2. Correlation between range-based and Christmas Bird Count-based values of community composition metrics based on categorical traits**. For the 351 species with complete distribution, trait and abundance data. For each of the two categorical traits considered – migratory behaviour and diet – maps were generated of community-weighted mean trait value (CWM; a, d, f, i), functional richness (FRICH; b, g) and functional divergence (FDIV; c, e, h, j). For the categorical traits, community-weighted mean was calculated as the proportion of birds in each of the trait classes. Observed values were calculated from recorded abundances at 68 evaluation Christmas Bird Count (CBC) sites. Modelled values were generated using four methods, with the best two shown here, as in Fig. 3. Lines represent y = x. Full results for all four methods are presented in Table S2 (below).


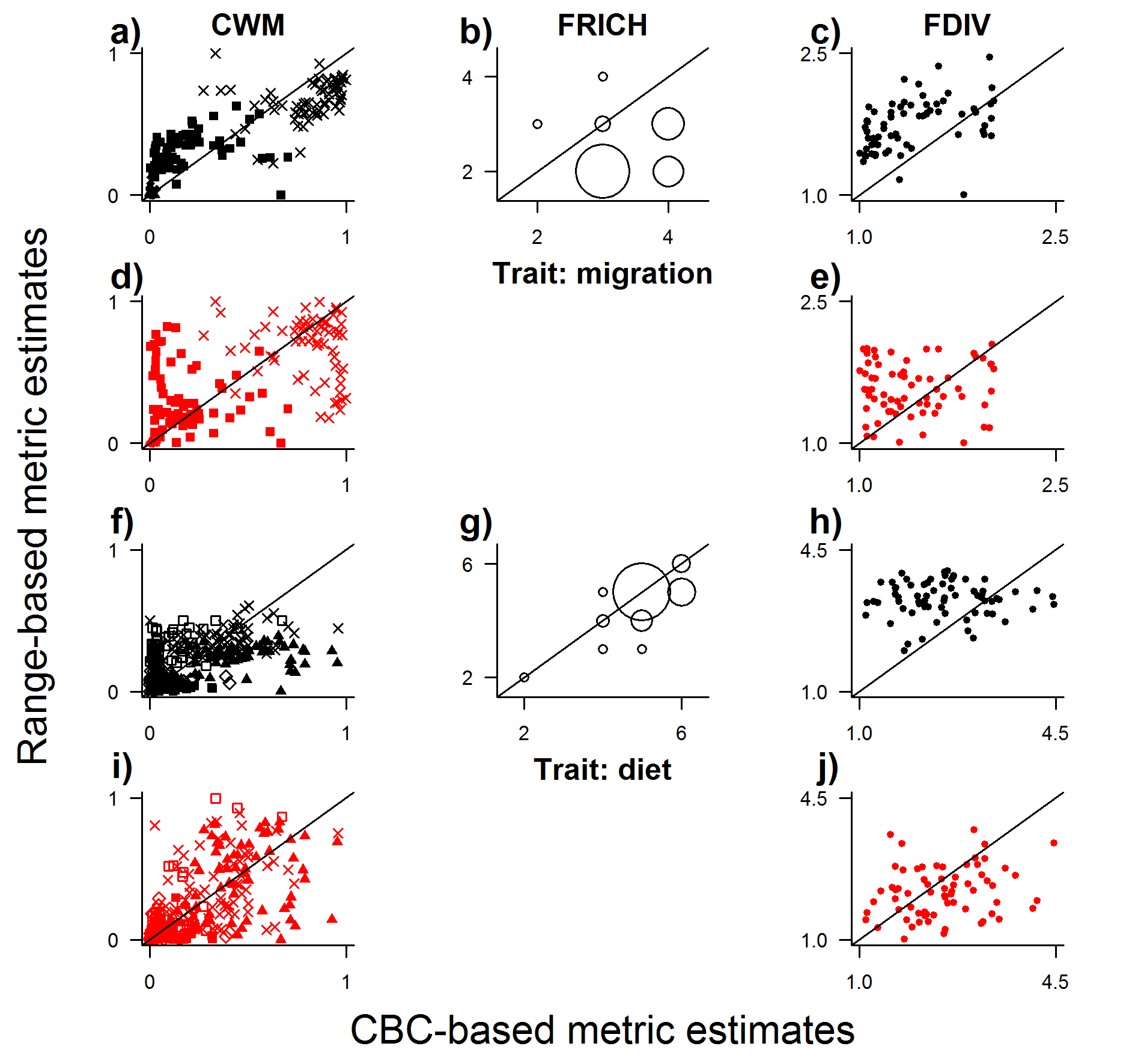


**Figure S3.** **Correlation between range-based and Christmas Bird Count-based values of community composition metrics based on all traits – body mass, generation length, migratory behaviour and diet – together**. For the 351 species with complete distribution, trait and abundance data. Maps were generated of functional richness (FRICH; a) and functional divergence (FDIV; b, c). Functional divergence was measured using the Rao index. Observed values were calculated from recorded abundances at 68 evaluation Christmas Bird Count (CBC) sites. Modelled values were generated using four methods, with the best two shown here, as in Fig. 3. Lines represent y = x. Full results for all four methods are presented in Table S2 (below).


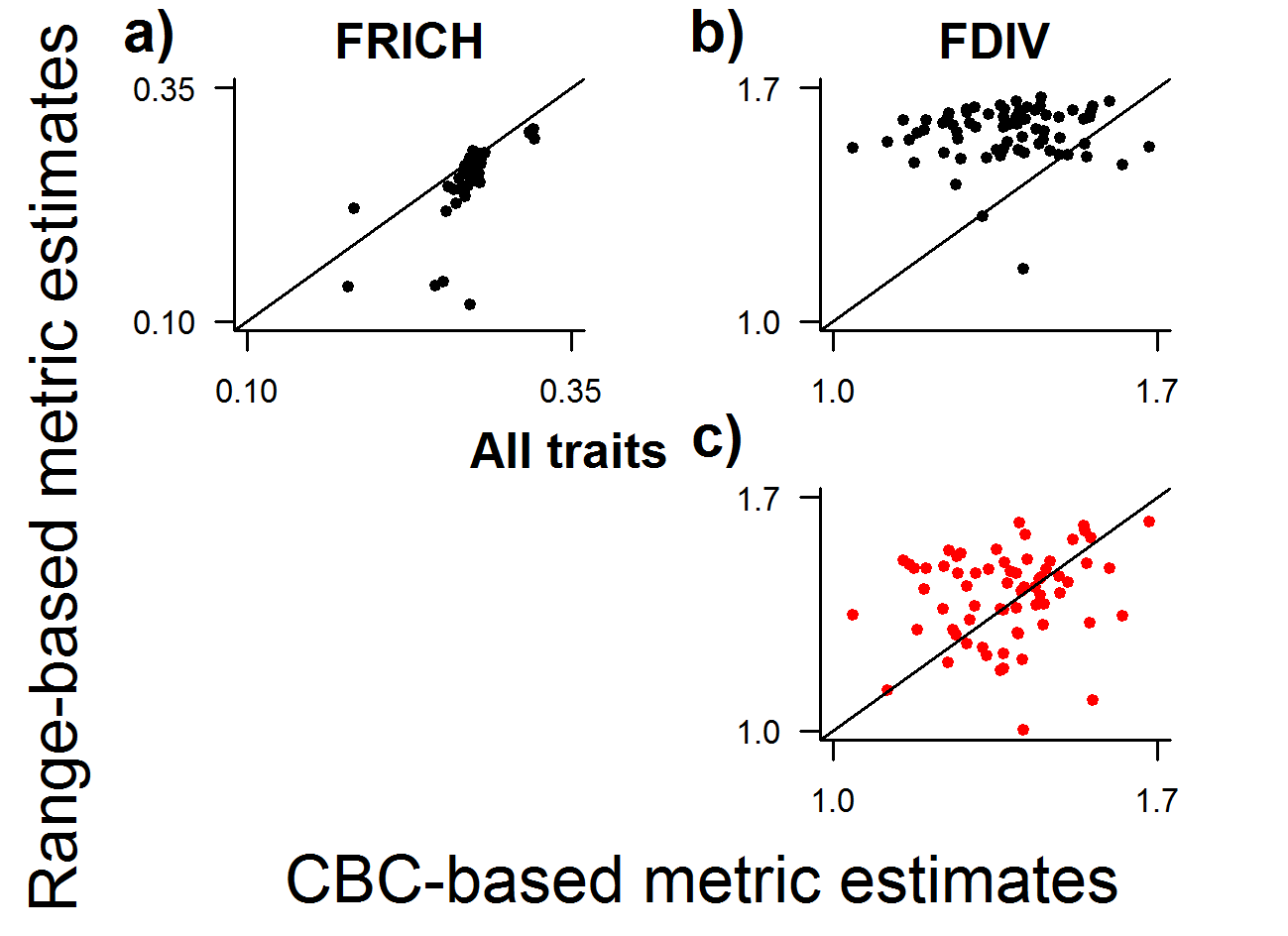


**Table 1. Full results of the fitted relationships between range-based and Christmas Bird Count-based estimates of** community composition for the 351 species with complete distribution, trait and abundance data.

| Trait | Metric | Method | Correlation | | Departure from unity slope | | |
| --- | --- | --- | --- | --- | --- | --- | --- |
|  |  |  | R^2^ | P | Slope | t | P |
| Mass | CWM | Range maps | **0.26** | **< 0.001** | 0.32 | 10.1 | < 0.001 |
|  |  | Total population | 0.17 | < 0.001 | **0.44** | **4.64** | < 0.001 |
|  |  | Mean of records | 0.12 | 0.0032 | 0.32 | 6.47 | < 0.001 |
|  |  | GAM models | 0.11 | 0.0059 | 0.4 | 4.33 | < 0.001 |
|  | FRICH | Range maps | **0.42** | **< 0.001** | **0.91** | **0.704** | 0.24 |
|  | FDIV | Range maps | 0.00018 | 0.91 | -0.0088 | 12.6 | < 0.001 |
|  |  | Total population | 0.0025 | 0.69 | -0.036 | 11.5 | < 0.001 |
|  |  | Mean of records | 0.0099 | 0.42 | -0.089 | 9.9 | < 0.001 |
|  |  | GAM models | **0.000075** | **0.94** | **0.012** | **5.98** | < 0.001 |
| Generation length | CWM | Range maps | **0.29** | **< 0.001** | 0.39 | 8.14 | < 0.001 |
|  |  | Total population | 0.24 | < 0.001 | **0.9** | **0.486** | 0.31 |
|  |  | Mean of records | 0.094 | 0.011 | 0.23 | 8.68 | < 0.001 |
|  |  | GAM models | 0.13 | 0.0033 | 0.31 | 6.82 | < 0.001 |
|  | FRICH | Range maps | **0.59** | **< 0.001** | **0.66** | **4.95** | < 0.001 |
|  | FDIV | Range maps | 0.049 | 0.07 | 0.12 | 13.2 | < 0.001 |
|  |  | Total population | 0.014 | 0.33 | 0.11 | 7.77 | < 0.001 |
|  |  | Mean of records | 0.02 | 0.25 | 0.11 | 9.39 | < 0.001 |
|  |  | GAM models | **0.098** | **0.0097** | **0.39** | **4.19** | < 0.001 |
| Migratory behaviour | CWM  (non-migratory) | Range maps | **0.058** | **0.048** | **0.16** | **10.7** | < 0.001 |
|  |  | Total population | 0.0091 | 0.44 | 0.14 | 4.72 | < 0.001 |
|  |  | Mean of records | 0.014 | 0.34 | 0.14 | 5.64 | < 0.001 |
|  |  | GAM models | 0.058 | 0.05 | -0.28 | 9.2 | < 0.001 |
|  | CWM  (nomadic) | Range maps | 0.23 | < 0.001 | **1.3** | **0.935** | 0.18 |
|  |  | Total population | **0.29** | **< 0.001** | 0.18 | 23.4 | < 0.001 |
|  |  | Mean of records | 0.26 | < 0.001 | 0.33 | 9.96 | < 0.001 |
|  |  | GAM models | 0.23 | < 0.001 | 0.24 | 13.8 | < 0.001 |
|  | CWM  (altitudinal migrants) | Range maps | 0.0009 | 0.81 | **0.04** | **5.82** | < 0.001 |
|  |  | Total population | 0.0035 | 0.63 | -0.0084 | 58.1 | < 0.001 |
|  |  | Mean of records | 0.0002 | 0.91 | 0.0016 | 71.7 | < 0.001 |
|  |  | GAM models | **0.0045** | **0.59** | 0.0067 | 80.3 | < 0.001 |
|  | CWM  (full migrants) | Range maps | **0.071** | **0.028** | **0.2** | **9.12** | < 0.001 |
|  |  | Total population | 0.011 | 0.4 | 0.15 | 4.76 | < 0.001 |
|  |  | Mean of records | 0.012 | 0.37 | 0.13 | 5.82 | < 0.001 |
|  |  | GAM models | 0.058 | 0.049 | -0.27 | 9.36 | < 0.001 |
|  | FRICH | Range maps | **0.14** | **0.0014** | **0.36** | **5.94** | < 0.001 |
|  | FDIV | Range maps | **0.16** | **< 0.001** | **0.36** | **6.39** | < 0.001 |
|  |  | Total population | 0.19 | < 0.001 | -0.43 | 13.1 | < 0.001 |
|  |  | Mean of records | 0.071 | 0.028 | -0.26 | 10.8 | < 0.001 |
|  |  | GAM models | 0.0017 | 0.74 | 0.036 | 8.91 | < 0.001 |
| Diet | CWM  (fruit) | Range maps | **0.15** | **0.0012** | 0.19 | 14.7 | < 0.001 |
|  |  | Total population | 0.097 | 0.0098 | 0.18 | 12.2 | < 0.001 |
|  |  | Mean of records | 0.11 | 0.0067 | 0.2 | 11.1 | < 0.001 |
|  |  | GAM models | 0.14 | 0.0015 | **0.27** | **8.84** | < 0.001 |
|  | CWM  (nectar) | Range maps | 0.79 | < 0.001 | 0.65 | 8.38 | < 0.001 |
|  |  | Total population | **0.8** | **< 0.001** | 0.67 | 7.97 | < 0.001 |
|  |  | Mean of records | 0.79 | < 0.001 | 0.37 | 27.5 | < 0.001 |
|  |  | GAM models | 0.64 | < 0.001 | **0.75** | **3.63** | < 0.001 |
|  | CWM  (other plant material) | Range maps | **0.11** | **0.006** | 0.17 | 14.2 | < 0.001 |
|  |  | Total population | 0.057 | 0.05 | 0.24 | 6.23 | < 0.001 |
|  |  | Mean of records | 0.09 | 0.013 | 0.2 | 10.3 | < 0.001 |
|  |  | GAM models | 0.11 | 0.0065 | **0.4** | **4.14** | < 0.001 |
|  | CWM  (invertebrates) | Range maps | **0.15** | **< 0.001** | 0.16 | 18.2 | < 0.001 |
|  |  | Total population | 0.081 | 0.018 | 0.31 | 5.33 | < 0.001 |
|  |  | Mean of records | 0.052 | 0.062 | 0.18 | 8.49 | < 0.001 |
|  |  | GAM models | 0.12 | 0.0039 | **0.42** | **4.06** | < 0.001 |
|  | CWM  (vertebrates) | Range maps | 0.2 | < 0.001 | 0.24 | 13.2 | < 0.001 |
|  |  | Total population | 0.012 | 0.37 | 0.08 | 10.5 | < 0.001 |
|  |  | Mean of records | 0.43 | < 0.001 | **0.53** | **6.26** | < 0.001 |
|  |  | GAM models | **0.52** | **< 0.001** | 1.1 | 0.954 | 0.17 |
|  | CWM  (mixed) | Range maps | 0.035 | 0.13 | 0.11 | 12.2 | < 0.001 |
|  |  | Total population | 0.0037 | 0.62 | -0.078 | 6.89 | < 0.001 |
|  |  | Mean of records | 0.0036 | 0.63 | 0.042 | 11 | < 0.001 |
|  |  | GAM models | **0.1** | **0.0093** | **0.31** | **5.92** | < 0.001 |
|  | FRICH | Range maps | **0.46** | **< 0.001** | **0.69** | **3.37** | < 0.001 |
|  | FDIV | Range maps | 0.000000017 | 1 | 0.000072 | 14.8 | < 0.001 |
|  |  | Total population | 0.0047 | 0.58 | -0.046 | 12.6 | < 0.001 |
|  |  | Mean of records | 0.002 | 0.71 | -0.024 | 15.6 | < 0.001 |
|  |  | GAM models | **0.048** | **0.075** | **0.18** | **8.2** | < 0.001 |
| All traits | FRICH | Range maps | **0.44** | **< 0.001** | **1.1** | **0.831** | 0.2 |
|  | FDIV | Range maps | 0.0048 | 0.58 | 0.044 | 12.2 | < 0.001 |
|  |  | Total population | 0.034 | 0.13 | -0.17 | 10.5 | < 0.001 |
|  |  | Mean of records | 0.021 | 0.23 | -0.12 | 11.3 | < 0.001 |
|  |  | GAM models | **0.037** | **0.12** | **0.2** | **6.3** | < 0.001 |
| Species richness | | Range maps | **0.84** | **< 0.001** | **0.96** | **0.822** | 0.21 |
| Total abundance | | Total population | 0.34 | < 0.001 | 0.34 | 11.2 | < 0.001 |
|  | | Mean of records | 0.47 | < 0.001 | 0.43 | 10 | < 0.001 |
|  | | GAM models | **0.56** | **< 0.001** | **0.71** | **3.68** | < 0.001 |

For each of the four traits considered (mean mass, generation length, migratory behaviour and diet) and for all traits together, we calculated community-weighted mean trait values (CWM), functional richness (FRICH) and functional divergence (FDIV). The overall fit of the relationship between mapped and observed community composition was measured using a correlation test (R^2^ and P-values reported). Departures from a fitted relationship of y = x (i.e. a slope of 1) were assessed using a t-test (slope, t and P-values reported). For each metric the results of the strongest correlation and the smallest departure from one are emboldened.
