# Supplementary material for: Mapping Functional Traits: Comparing Abundance and Presence-Absence Estimates at Large Spatial Scales
Source: PLoS One. 2012 Aug 31;7(8):e44019. doi: 10.1371/journal.pone.0044019 (PMC3432103; doi:10.1371/journal.pone.0044019)
Supplement: Appendix S3 — Frequency distributions of species with respect to body mass and generation length. (DOC) [file pone.0044019.s003.doc]

**Appendix S3. Frequency distributions of species with respect to body mass and generation length.**

**Figure S4. Frequency distributions of species with respect to body mass and generation length.** Both traits were divided into ten equal-sized bins in log space.
